# Biomimetic Hydroxyapatite on Graphene Supports for Biomedical Applications: A Review

**DOI:** 10.3390/nano9101435

**Published:** 2019-10-10

**Authors:** Gang Wei, Coucong Gong, Keke Hu, Yabin Wang, Yantu Zhang

**Affiliations:** 1 College of Chemistry & Chemical Engineering, Yan’an University, Yan’an 716000, China; 18329903132@163.com (K.H.); ybw@yau.edu.cn (Y.W.); 2 Faculty of Production Engineering, University of Bremen, D-28359 Bremen, Germany; ccgong@uni-bremen.de

**Keywords:** graphene, hydroxyapatite, biomimetic synthesis, nanohybrids, biomedical applications

## Abstract

Hydroxyapatite (HA) has been widely used in fields of materials science, tissue engineering, biomedicine, energy and environmental science, and analytical science due to its simple preparation, low-cost, and high biocompatibility. To overcome the weak mechanical properties of pure HA, various reinforcing materials were incorporated with HA to form high-performance composite materials. Due to the unique structural, biological, electrical, mechanical, thermal, and optical properties, graphene has exhibited great potentials for supporting the biomimetic synthesis of HA. In this review, we present recent advance in the biomimetic synthesis of HA on graphene supports for biomedical applications. More focuses on the biomimetic synthesis methods of HA and HA on graphene supports, as well as the biomedical applications of biomimetic graphene-HA nanohybrids in drug delivery, cell growth, bone regeneration, biosensors, and antibacterial test are performed. We believe that this review is state-of-the-art, and it will be valuable for readers to understand the biomimetic synthesis mechanisms of HA and other bioactive minerals, at the same time it can inspire the design and synthesis of graphene-based novel nanomaterials for advanced applications.

## 1. Introduction

Hydroxyapatite (HA) is one of the important inorganic components of the human teeth and bone, and it has shown wide applications in the fields of biomaterials science [1], tissue engineering [2], biomedicine [3], energy and environmental science [4,5,6], and analytical science [7] due to its simple preparation, low-cost, and high biocompatibility. Previously, several main synthesis strategies, such as hydrothermal synthesis [8], electrochemical deposition [9], chemical vapor deposition [10], in-situ biomimetic synthesis [11], and biomimetic mineralization [12,13], have been demonstrated to create various HA nano-/micro-materials with different structures and properties, in which the biomimetic strategies exhibited great advantages comparing to other synthesis methods. For instance, the reaction condition of biomimetic synthesis is very mild and easy to control, the biomimetic HA-based nanomaterials showed higher stability and biocompatibility, and the biomimetic HA materials exhibited highly ordered structures and architectures by parameter adjustment of mineralization.

However, it is difficult to apply the pure HA for bone tissue engineering and biomedical implant due to its relative weak toughness and tensile strength. To solve this problem, the combination of HA with other kinds of bioactive tough materials, such as Ti alloys [14], polymer matrix [15], self-assembled peptide/protein nanofibers [16,17], carbon materials (carbon nanotubes and graphene) [18,19], and others [20,21], have been performed to improve the mechanical properties of HA-based materials. It is obvious that the introduction of these reinforcing materials to HA extended the potential applications of HA in biomedical fields.

Graphene has attracted great attention in the fabrication of various novel nanomaterials due to its unique two-dimensional (2D) structure and innovative biological, electrical, mechanical, thermal, and optical properties [22,23,24,25,26]. Graphene revealed several advantages as the reinforcing material for promoting the biomedical applications of HA. First, it provides a 2D flat support for the nucleation and growth of HA with ordered structure. Secondly, graphene-based materials, especially graphene oxide (GO) and reduced GO (RGO), have a large number of functional groups on the surface of graphene, which can be easily modified for the formation/binding of HA. Thirdly, graphene has been reported to have good biocompatibility and can mediate bone repair and regeneration. Fourthly, graphene has been proven to have a strong mechanical strength, high elasticity, and good flexibility. Thanks to the development of both graphene synthesis and biomimetic mineralization techniques, a lot of studies have been carried out to create HA on graphene supports to inspire the potentials of the synthesized hybrid materials in biomedical engineering [19,27]. For example, Wang and co-workers reported the synthesis of GO-incorporated collagen/HA composites for bone repair applications [27]; Murugan et al. demonstrated the mineralization of HA on GO/carbon nanofibers (CNFs) for anti-bacterial and anti-osteoblast cells applications [28]; Wei and co-workers reported the peptide/protein nanofibers-induced biomimetic synthesis of HA on GO supports for cell adhesion and growth [19,29].

**Figure 1:**
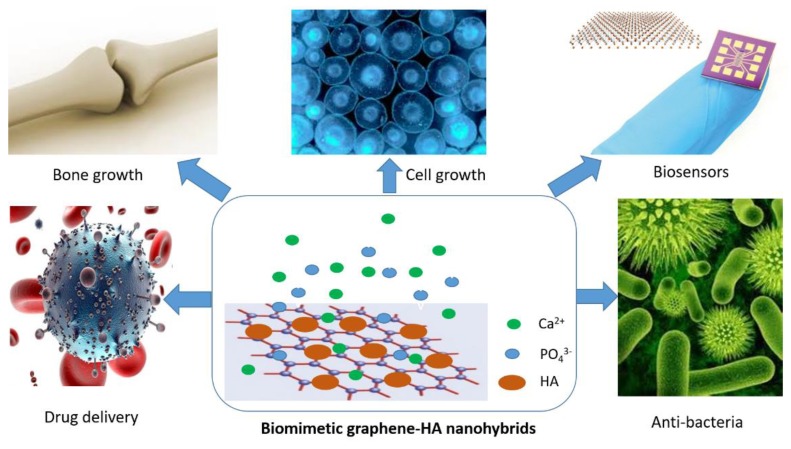
Biomedical applications of biomimetic graphene-HA materials.

Previously, a few review papers on the biomimetic graphene-HA hybrid composites for biomedical applications have been reported [30,31,32]. For instance, Li and co-workers presented the formation of graphene-HA composites for orthopedic applications [30] and Basirum et al. provided an overview on the preparation of graphene-HA nanocomposites for bone graft substitute applications [31]. Although great achievements have been made, there are still other spaces that could be extended to promote and inspire the development of this research field. Here, we would like to contribute an overview of recent advancement in the biomimetic synthesis of HA on graphene supports (GO and RGO) for biomedical applications. More focus on the biomimetic synthesis methods of HA and HA on graphene supports are introduced in Part 2 and 3, and then the biomedical applications of biomimetic graphene-HA in drug delivery, cell growth, bone regeneration, biosensors, and antibacterial test are demonstrated and discussed in Part 4 in great detail, as shown in Figure 1. We believe that this work is the state-of-the-art and it will be valuable for readers to understand the biomimetic synthesis of HA and other bioactive minerals, at the same time as inspiring the design and synthesis of graphene-based novel materials for advanced applications.

## 2. Biomimetic Strategies of HA

**Figure 2:**
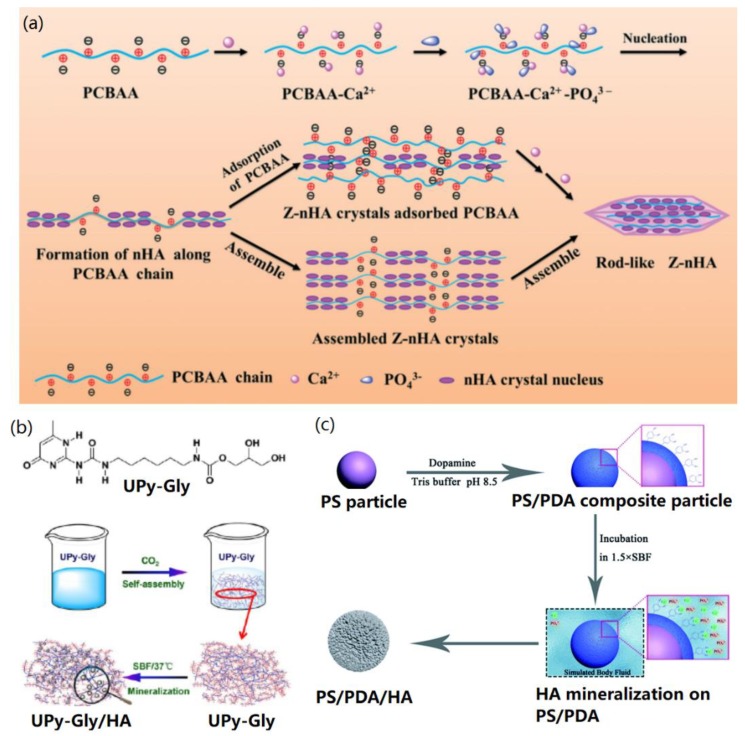
Biomimetic synthesis strategies of HA: (**a**) In-situ biomimetic chemical synthesis of HA in the presence of Ca^2+^ and HPO_4_^2−^. Reprinted with the permission from [35]. Copyright 2018 Royal Society of Chemistry. (**b**) HA biomineralization in simulated body fluid (SBF). Reproduced with the permission from [36]. Copyright 2017 American Chemical Society. (**c**) HA biomineralization in 1.5×SBF. Reproduced with the permission from [37]. Copyright 2016 Royal Society of Chemistry.

Inspired by nature, biomimetic strategies have been widely utilized for the facial synthesis of various bioactive nanomaterials without using high temperature, high pressure, and hazard chemicals [33,34]. Typically, various HA structures (nanoparticles, nanoneedles, nanowires, and porous microspheres) can be biomimetic synthesized via in-situ biomimetic chemical synthesis and biomimetic mineralization in simulated body fluid (SBF) solution, as shown in Figure 2 [35,36,37].

It is possible to directly create HA within a short term (about 30 min) through in-situ biomimetic chemical synthesis, in which the presence of diluted Ca^2+^ and HPO_4_^2−^ mediated the nucleation and growth of HA crystals on various inorganic or organic templates with the surface groups of –COOH, –NH_2_, and –OH [38]. By using this method, for the first time, Hartgerink et al. reported the biomimetic synthesis of HA on self-assembled peptide nanofibrils [39]. It was found that the crystallographic c axes of HA were aligned along the long axes of peptide nanofibrils, which is similar to the alignment between collagen fibers and HA in human bone. Recently, more studies have been carried out for in-situ biomimetic synthesis of HA by using biomacromolecules [40] and polymers [35,41] as nucleation supports. In a typical study, Xu and co-workers demonstrated the in-situ biomimetic synthesis of nanoscale HA crystals in the presence of a zwitterionic polymer poly(3-carboxy-N,N-dimethyl-N-(3′-acrylamidopropyl) propanaminium inner salt) (PCBAA) [35], and found that PCBAA was the key factor to modulate the nucleation and growth of HA, as shown in Figure 2a. The negative –COOH group of PCBAA first adsorbed Ca^2+^ along the polymer chain by electrostatic and coordination (Ca^2+^–CONH–) interactions when CaCl_2_ was added, and then the addition of (NH_4_)_2_HPO_4_ solution promoted the deposition of PO_4_^3−^ ions onto the polymer chain via their electrostatic interactions with –R_3_N^+^ groups and Ca^2+^. Due to the PCBAA-mediated nucleation and growth, HA nanocrystals could be formed quickly (0.5 h). With the increasing mineralization period to several days, HA nanocrystals were further adsorbed and assembled to form rod-like HA crystals. Their study provided new ideas for studying the mechanism of protein-inspired biomimetic mineralization due to the similar chemical structure of PCBAA to proteins.

In order to mimic the mineralization of HA in biological environment, SBF was developed as an organic-free salt buffer to replace the in-situ chemical synthesis [42]. With this strategy, the structure and Ca/P ratio of the mineralized HA (or apatite) can be adjusted by changing the mineralization period (from hours to days). In addition, to mimic the biological ion condition precisely, it is necessary to replace the used SBF solution daily. Previously, Su et al. demonstrated the protein-mediated biomimetic mineralization of HA on electrospun poly (ε-caprolactone) (PCL) nanofibers [43], and found that the biocompatible bone protein 2 (BMP2) could improve the HA mineralization efficiency obviously. In another case, Li and co-workers reported the biomimetic mineralization of bone-like HA by incubating supramolecular porous fiber networks in SBF solution [36], as shown in Figure 2b. In their study, 2-ureido-4[1H]-pyrimidone-modified glycerol molecules (UPy-Gly) were self-assembled into a porous nanofiber network, which was utilized as a support for HA mineralization. It was found that apatite nuclei were first created in the pores of substrate and then HA crystals were formed after 7 days incubation in SBF. The formed HA crystals exhibited high elasticity as well as bone-like structure and properties, showing potential applications for bone tissue implantation and regeneration.

To accelerate the nucleation and mineralization process, the buffer solution with 1.5-folder ionic strength to SBF (named as 1.5× SBF) has also been widely utilized for biomimetic synthesis of HA crystals [12,18,21,37,44]. For example, previously, Wei et al. demonstrated that proteins could mediate the quick biomimetic mineralization of HA in 1.5× SBF (about 7 days) [12,18]. Cui and co-workers reported the biomimetic mineralization of HA on polydopamine (PDA)-functionalized polystyrene (PS) particles [37] by using 1.5× SBF. As show in Figure 2c, PDA on the surface of PS particles adsorbed the mineral ions and anchored the nucleation and growth of HA nanocrystals on the surface of PS particles, as indicated in Figure 2c. It was found that HA nanocrystals were formed after incubating in 1.5× SBF for three days. In addition, supersaturated SBF solutions, such as 5× SBF [45,46] and 10× SBF [47] have also been utilized to obtain the nucleation and crystallization of HA nanocrystals on various mineralization substrates in a few days. All the above studies indicate that biomimetic strategies play important roles in the synthesis and biomedical applications of HA-base materials. However, the constitute, structure, and properties of mineralized HA are ascribed to the type and concentration of ions in the mineralization environment.

## 3. Graphene-Supported Biomimetic Synthesis of HA

In this section, we would like to present the biomimetic synthesis strategies of HA on both graphene supports and graphene-based nanohybrids.

### 3.1. Graphene for Biomimetic HA

The surface groups (such as –COOH, –OH, –NH_2_) on support materials are crucial for the biomimetic synthesis of HA crystals as introduced above in the cases in Part 2. To overcome the problem of hard mineralization towards pure graphene support, GO and chemically modified GO surfaces were utilized for in-situ biomimetic synthesis and mineralization [48,49,50,51,52].

**Figure 3:**
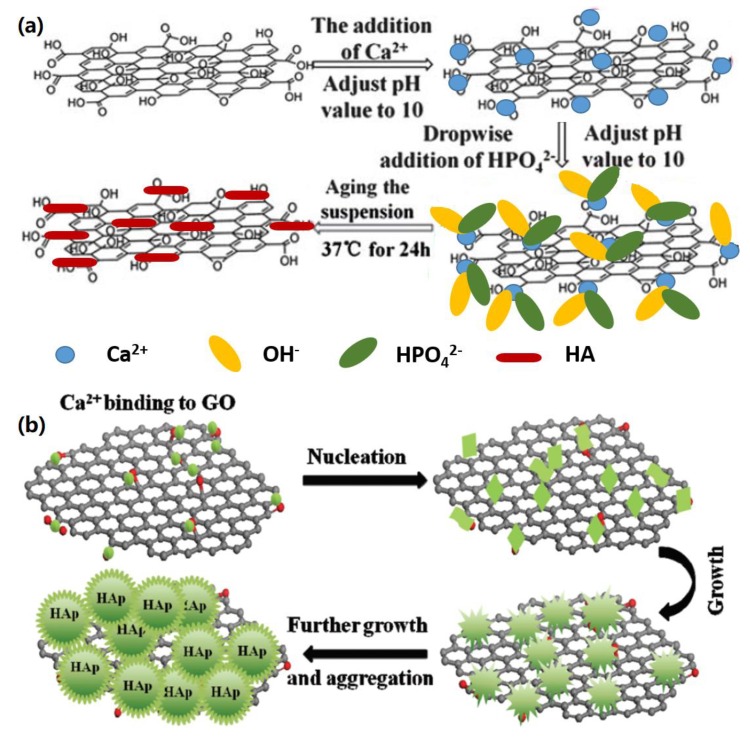
Biomimetic synthesis of HA on pure graphene supports (**a**) HA mineralization on GO in the presence of Ca^2+^ and HPO_4_^2−^. Reproduced with the permission from ref. [48]. Copyright 2013 Royal Society of Chemistry. (**b**) HA synthesis on GO in SBF solution. Reprinted with the permission from ref. [51]. Copyright 2014 Royal Society of Chemistry.

Li et al. reported the in-situ biomimetic synthesis of HA on pristine GO surface [48]. As shown in Figure 3a, when Ca^2+^ ions were added onto the GO surface under pH 10, the oxygen-active groups of GO adsorbed Ca^2+^ via the electrostatic interactions, and subsequent addition of HPO_4_^2−^ into the mineralization system caused the formation of nanoscale HA crystals. The reaction period of 24 h promoted the formation of nanorod-like HA crystals. In a similar case, Tang and co-workers reported the preparation of HA nanoplates on GO support through the in-situ biomimetic synthesis method [49]. Maser and co-workers introduced a novel rapid in-situ biomimetic synthesis method to create nanocrystalline HA on GO support [50], in which CaCO_3_ and H_3_PO_4_ were mixed with GO, and NH_4_OH solution was added under pH 10. It was found that HA crystals could be formed within 30 min.

In addition to in-situ biomimetic synthesis, the biomimetic HA mineralization of GO can be achieved in the SBF system [51,52]. For instance, Wen et al. demonstrated a simple biomimetic strategy to fabricate 3D hierarchical GO-HA nanocomposites by incubating GO in a modified SBF solution [51]. As indicated in Figure 3b, Ca^2+^ ions were first adsorbed onto GO surface in SBF by the electrostatic interactions with oxygen-active groups and then small apatite cyrstals were formed in a short mineralization period. After further nucleation and crystal growth, nanoscale and microscale HA crystals were formed by adjusting the mineralization period. The formed GO-HA nanocomposites with this method exhibited a high adsorption and ion-exchange capacity, and therefore, can be potentially used for water purification applications. In another study, Gao et al. reported the biomimetic synthesis of HA on GO-coated Mg alloy in SBF [52]. It was found that GO greatly promoted the formation of dense HA crystals on Mg alloy, which improved the anti-corrosion resistance of materials greatly.

### 3.2. Graphene-Based Nanohybrids for Biomimetic HA

In addition to pure GO, chemically modified GO, and RGO, other composite materials such as GO and RGO-based nanohybrids could be potential supports for biomimetic synthesis of HA crystals. Previously, various building blocks, including apatite [53], small molecules [54,55,56], biomacromolecules [50,57], self-assembled peptide/protein nanostructures [19,29,58], and cells [59,60] have been widely used to create graphene-based nanohybrids for biomineralization of HA.

For instance, Fan et al. investigated the modification of carboxylated GO with casein phosphopeptide, and further studied the created graphene-peptide biocomposites for HA biomineralization in SBF [54]. It was found that casein phosphopeptide could not only improve the bioactivity and biocompatibility of GO, but also promote the nucleation and growth of HA crystals on GO support. Li and co-workers demonstrated that chitosan (CS)-modified GO could serve as an excellent template for biomimetic synthesis of HA [48], which provided new ideas for creating functional GO-HA materials for bone tissue engineering and bio-coating applications.

**Figure 4:**
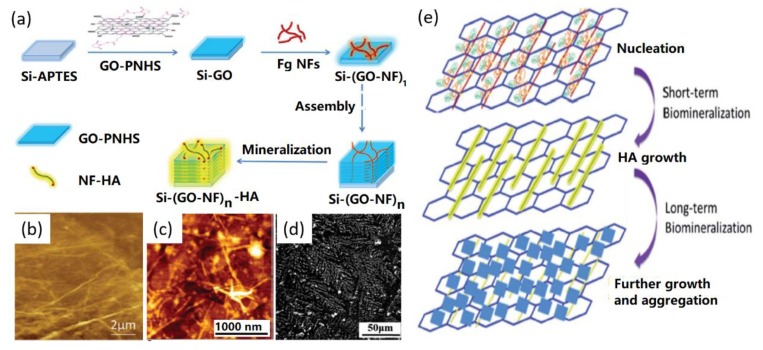
GO-nanofibers (NFs) for HA biomimetic mineralization: (**a**) Synthesis process, (**b**) AFM image of GO-NFs, (**c**) AFM image of GO-NFs after mineralization for 2 h, (**d**) SEM image of GO-NFs after 14 days mineralization, and (**e**) possible biomimetic synthesis mechanism of HA on GO-NFs. Reproduced with the permission from ref. [29]. Copyright 2014 Royal Society of Chemistry.

To promote the formation of bioactive and functional HA nano/micro crystals on GO supports, Wang and co-workers introduced self-assembled protein [29] and peptide nanofibers (NFs) [19], as well as peptide nanosheets [58] to modify GO for further biomineralization of HA. For instance, previously, they utilized a layer-by-layer assembly method to create three-dimensional (3D) GO-fibrinogen nanofibers (GO-NFs) nanohybrids [29], which were further mineralized in SBF solution to form 3D bio-scaffolds (Figure 4a). After short-term mineralization (a few hours), HA nanocrystals with a diameter of several nm were created on the GO-NFs (Figure 4b) to form GO-NF-HA nanohybrids (Figure 4c), while the long-term mineralization (two weeks) caused the formation of HA microspheres with a diameter of a few µm (Figure 4d) on GO surface. Through multi-characterizations of mineralized HA, the potential nucleation and growth mechanism of HA biomineralization on GO-NFs nanohybrids was proposed, which provides new guidance for designing and synthesizing functional reinforced biomaterials for biomedical applications.

**Figure 5:**
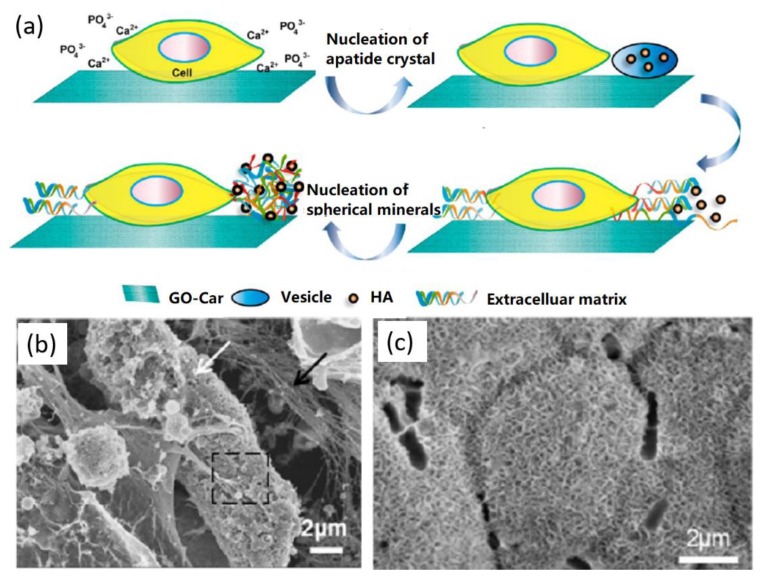
Cell-mediated biomimetic formation of HA on GO surface: (**a**) biomimetic formation mechanism of HA by cell. (**b**,**c**) Biomimetic synthesis of HA with 14 days mineralization of GO-Car (**b**) with and (**c**) without MC3T3-E1 cells. Reproduced with the permission from [59]. Copyright 2014 American Chemical Society.

Cells can also mediate the biomimetic mineralization of HA on graphene supports. In a typical study, Liu and co-workers reported the MC3T3-E1 cell-mediated biomimetic HA formation by using carrageenan (Car)-functionalized GO (GO-Car), as shown in Figure 5a [59]. Their results revealed that Car facilitated the attachment of cells and the GO-Car surface promoted the binding of Ca^2+^ ions and the nucleation of HA, which was first nucleated within the cell vesicles and then the growth of HA crystals broken the vesicles and exposed to the extracellular fluid. Finally, individual HA minerals consisting of calcium phosphate and collagen fibers were formed. The SEM characterizations indicated that minerals with a microporous structure and 3D channels were formed on the GO-Car support without cells after 14 days mineralization (Figure 5b), while the introduction of cells to the GO-Car support caused the formation of the complex of organic bundles and embedded calcium phosphate (Figure 5c). In another similar study [60], Cheng et al. presented the MC3T3-E1 cell-mediated biomimetic HA mineralization on the PDA-modified GO. Their study further proved that MC3T3-E1 cells exhibited higher cellular activities compared to both bare glass and GO substrates, revealing their potential applications for bone tissue regeneration.

Based on the above case studies, it can be concluded that the functionalization (especially biological modification) of graphene supports is the most effective strategy to create biomimetic graphene-HA materials, which showed controllable structure, high mechanical properties, and high biocompatibility that suitable for biomedical applications.

## 4. Biomedical Applications

In this section, the biomedical applications of biomimetic graphene-HA materials in drug delivery, cell culture, bone repair and growth, biosensors, and anti-bacteria fields are introduced and discussed further.

### 4.1. Drug Delivery

In recent years, great interest has been focused on the drug delivery and release due to increasing demands for cancer therapy [61]. Graphene can be served as a novel 2D biocompatible platform for drug delivery, which was attributed to special adsorption between GO and biomolecules and drugs via the non-covalent interactions [62]. Although these biomolecules, including drugs (such as doxorubicin (DOX)), glucose oxidase (GOx), horseradish peroxidase (HRP), lysozyme, and proteins (such as bovine serum albumin (BSA)), can be immobilized onto the GO surface through non-covalent interactions, some factors, such as the pH-dependent delivery and the release of drugs and biomolecules, the capacity of drugs, toxicity, and stability of delivery materials, should be further studied.

To introduce biomolecules into the drug delivery systems, GO-HA nanocomposites have been widely applied in drug delivery due to their unique affinity with biomolecules with a large adsorption capacity and pH-controlled release in various biomedical applications [63,64]. For example, Yao and co-workers reported a facile in-situ synthesis method to build GO-HA hybrids as drug carriers by using GO and creatine phosphate disodium (CPDS) as dual templates [65]. In their study, CPDS salt served as a phosphorus source and nucleation site for the mineralization of calcium phosphate and HA particles. The synthesized HA particles showed bubble-like aggregated shape with a wall thickness of about 300 nm and a hollow structure with a diameter range from 500 nm to 2 μm. The drug delivery-release behavior was also investigated by using ibuprofen as a drug model and the results showed that the sustained release capacity of GO-HA hybrids was better than pure HA particles due to the hierarchically and flower-like structure of GO-HA hybrids.

**Figure 6:**
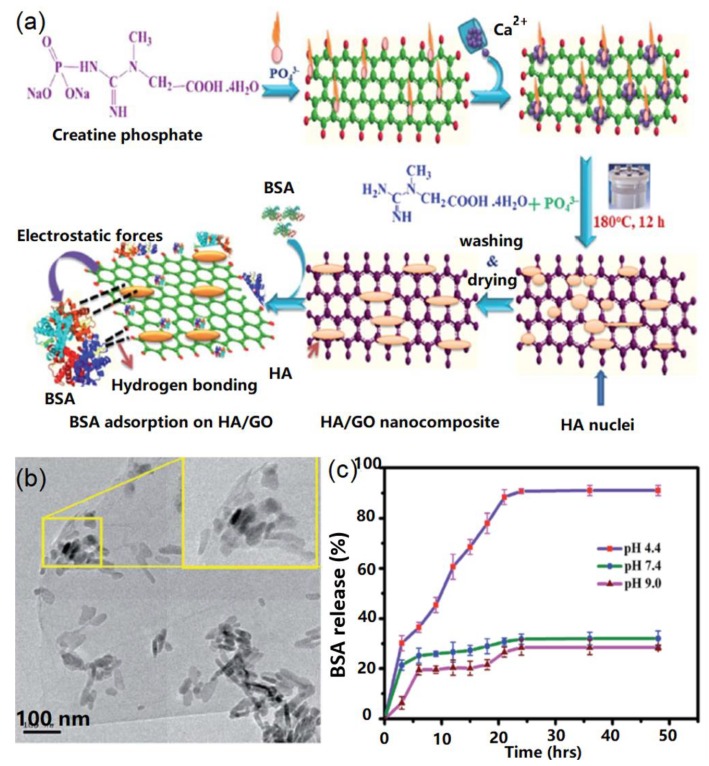
Biomimetic graphene-HA nanohybrids for drug delivery: (**a**) Synthesis of HA and drug delivery mechanism; (**b**) TEM image of graphene-HA nanohybrids; (**c**) drug delivery tests. Reproduced with the permission from ref. [66]. Copyright 2017 Royal Society of Chemistry.

In another case, Bharath and co-authors developed a novel biomimetic approach to synthesize HA nanorods on the GO surface by using CPDS as phosphorus source for drug delivery [66], as shown in Figure 6. CPDS was first absorbed onto GO surface via the electrostatic interactions between carboxyl and amino groups. Subsequently, Ca^2+^ ions were introduced to bind with the remaining epoxy and hydroxyl functional groups of GO sheets through the electrostatic interactions. The PO_4_^3−^ ions were slowly released from the CPDS molecules and then reacted with Ca^2+^ ions to form HA nuclei through the electrostatic interactions (Figure 6a). The obtained transmission electron microscope (TEM) results showed that the HA nanorods were uniformly dispersed on the GO surface with an average length of 20–85 nm and a diameter of 20 nm (Figure 6b). In addition, the adsorption and release of drugs were estimated by using BSA as a model and the results indicated that the BSA adsorption was up to 97.5% when the adsorption occurred at a neutral condition. The BSA release was up to 91% after 50 h at pH 4.4 compared with 32% and 38% release at pH 7.4 and 9.0, respectively (Figure 6c). Their study indicated that the biomimetic GO-HA nanohybrids had a high drug loading efficiency, a good biocompatibility, and excellent pH sensitivity for the drug delivery system.

GO can also be modified with biomolecules, including peptides, proteins, DNA, and CS to promote the nucleation and growth of HA crystals. For instance, Gholibegloo et al. prepared carnosine-conjugated GO hybrids via the covalent interactions between 1-ethyl-3-(3-dimethylaminopropyl) carbodiimide (EDC) and N-hydroxysuccinimide (NHS) reagents to promote the nucleation and growth of HA on multi-functionalized GO surface (GO-carnosine/HA) [67]. Improved indocyanine green (ICG) loading was obtained and the multi-functional nanocarriers had a high loading capacity of 57.52% and long-term stability.

### 4.2. Cell Culture

Biomimetic graphene-HA nanohybrids are excellent scaffolds for the adhesion and growth of various cells due to their strong mechanical properties, high biocompatibility, and unique structures [68,69,70,71,72].

Previously, Kim et al. demonstrated that biomimetic HA can be formed on GO surface for improving the adhesion and proliferation of osteoblast cells [73]. To enhance the bioactivity of GO-HA based biomaterials for cell culture, bioactive protein and peptide have been incorporated for creating novel functional hybrid materials [19,29,69,74,75]. For instance, Wang et al. found that the addition of protein and peptide NFs onto GO supports could promote the biomimetic synthesis of HA nano/micro crystals for enhanced cell culture [19,29]. Nair and co-workers demonstrated the synthesis of GO that modified with gelatin-HA crystals, which exhibited enhanced osteogenic adhesion and differentiation of human stem cells [74].

**Figure 7:**
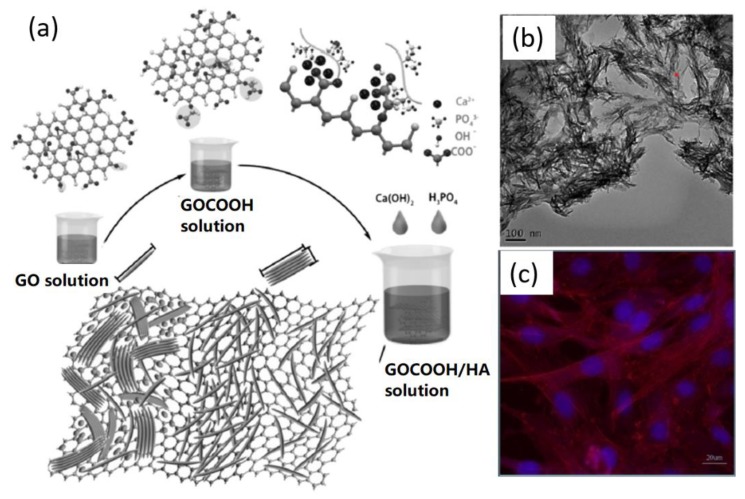
Biomimetic GO-HA nanohybrids for cell adsorption and growth: (**a**) Biomimetic synthesis of HA on GO-COOH substrate; (**b**) TEM image of GO-HA nanohybrids, and (**c**) Fluorescence image of GO-HA/silk fibroin (SF) scaffold after cell growth. Reproduced with the permission from ref. [75]. Copyright 2017 Elsevier Ltd.

Recently, Wang et al. demonstrated the -COOH group-medicated biomimetic synthesis of GO/HA scaffolds (Figure 7a), which could be further utilized to conjugate with silk fibroin (SF) to improve the cell culture performance [75]. Their results indicated that the –COOH group on GO surface contributed great effects to the biomimetic formation of HA crystals on GO. HA nanorods with a length of 80–120 nm and a width of 12 nm were synthesized, as indicated in the TEM image of Figure 7b. The cell test experiments revealed that the designed GO-HA/SF scaffolds stimulated the adhesion and proliferation of mouse mesenchymal stem cells (Figure 7c).

By using the mineralization of polymers, biomimetic GO-HA nanocomposites have also been utilized for cell culture [76,77]. For instance, Ramani and Sastry reported that cellulose was helpful for reinforcing the biomimetic formation of HA on GO support, and the fabricated GO-cellulose-HA scaffold was highly bioactive for promoting the adhesion and growth of both MG-63 and NIH-3T3 cells, making the designed biomimetic material a very good candidate for in vitro osteoinductive application [77].

### 4.3. Bone Repair and Regeneration

Due to the high potential of HA for bone tissue engineering and the strong mechanical strength of graphene support, biomimetic graphene-HA materials exhibited wide applications in the fields of bone repair and regeneration [78,79,80].

Wang et al. reported collagen (Col)-mediated biomimetic synthesis of HA on GO support for the formation of bioactive Col/GO-HA composites, which exhibited improved hydrophilic and mechanical properties, as well as the ability to promote the bone repair by testing the osteoblastic cells [27]. Besides osteoblastic cells, other cells, such as MC3T3-E1, human mesenchymal stem cells, and osteosarcoma play important roles in the bone growth, repair, and regeneration [81,82,83,84,85]. For instance, Zhang and co-workers presented the biomineralization of HA crystals with a size of tens nm on 3D graphene foams (GFs) in 10× SBF containing 10 mM HCO_3_^−^ ions [82]. The mesenchymal stem cells growth test indicated that the created 3D GF-HA materials exhibited faster osteogenic commitment and stronger osteogenic differentiation than HA materials, revealing their promising application for bone regeneration. In another case, Liu and co-workers reported a facile gelatin-mediated mineralization of HA on GO support, which showed higher cell adhesion, proliferation, and alkaline phosphatase activity compared to GO and glass surface [85]. The in vitro osteogenic differentiation with MC3T3-E1 cells proved the suitability of the fabricated GO-gelatin-HA nanohybrids for bone regeneration and surgery.

**Figure 8:**
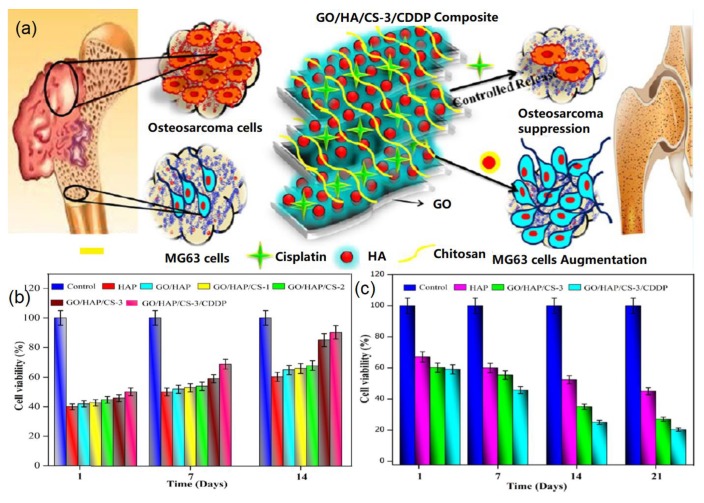
GO-HA nanohybrids for bone regeneration: (**a**) Bone regeneration mechanism, (**b**) MG63 cell viability on various samples, and (**c**) A549 cancer cell viability on different samples. Reproduced with the permission from [83]. Copyright 2018 American Chemical Society.

In addition, biomimetic graphene-HA nanohybrids can be utilized as regeneration medicines for bone repair [83,86]. For instance, Sumathra et al. reported a novel GO-HA based regeneration medicine tool for osteosarcoma-affected bone regeneration [83]. In their study, HA crystals were first biomineralized on GO surface and CS was then conjugated onto the formed GO/HA hybrids to create GO/HA/CS composites. It was found that the fabricated GO/HA/CS composites could load an anticancer drug, cisplatin (CDDP), to act as a promising tool for bone tissue engineering applications, as indicated in Figure 8a. The quantification analysis of cell viability of MG63 osteoblast-like cells (Figure 8b) and A549 cancer cells (Figure 8c) indicated that the synthesized GO/HA/CS/CDDP composites exhibited promising performances for not only promoting the growth of MG63 osteoblast-like cells, but also killing cancer cells (A549) and replacing the bone-cancer-affected tissues with fresh-grown healthy ones. This study provides a novel strategy to achieve in multi-functional applications of biomimetic graphene-HA materials. In another study, Zhang et al. designed a free-standing flexible membrane based on biomimetic hexagonal bars of HA on mesoporous graphene/single-walled carbon nanotubes (MG/SWCNT) hybrid membrane [86]. The fabricated MG/SWCNT-HA membrane promoted the adhesion and proliferation of human fetal osteoblast osteoprogenitor cells and enhanced in vitro biomineralization, showing great promise as regeneration medicine for spine fusion, bone repair, and restoration of tooth enamel.

### 4.4. Biosensors

Due to its unique properties, such as an excellent biocompatibility and a good adsorption ability, HA has become an attractive material for the fabrication of biosensors [87].

Previously, Pang and co-workers developed a simple and sensitive method for detecting luteolin by using graphene nanosheets (GNs)-HA composite as modified electrode materials [88]. In their study, the fabricated GNs-HA modified glassy carbon electrode (GCE) exhibited excellent electrocatalytic activity in the redox process of luteolin compared with GNs-GCE electrode, attributing to the enhanced electron transfer and electrocatalysis toward luteolin. In another case, an electrochemical sensor based on GO-HA composites-modified GCE was fabricated for simultaneous determination of 4-aminophenol (4-AP), uric acid (UA), and nitrite ions (NO^2−^) by cyclic voltammetry (CV) and square wave voltammetry measurements [89]. They found that the formed GO-HA nanocomposite exhibited synergistic effects with high sensitivity, low detection limits, good stability, and high reproducibility. The fabricated GO-HA hybrids-based biosensors showed a dynamic linear detection range of 0.1–425, 1–1000, and 3–950 μM with detection limits of 0.29, 0.03, and 0.025 μM for 4-AP, UA, and NO^2−^, respectively.

**Figure 9:**
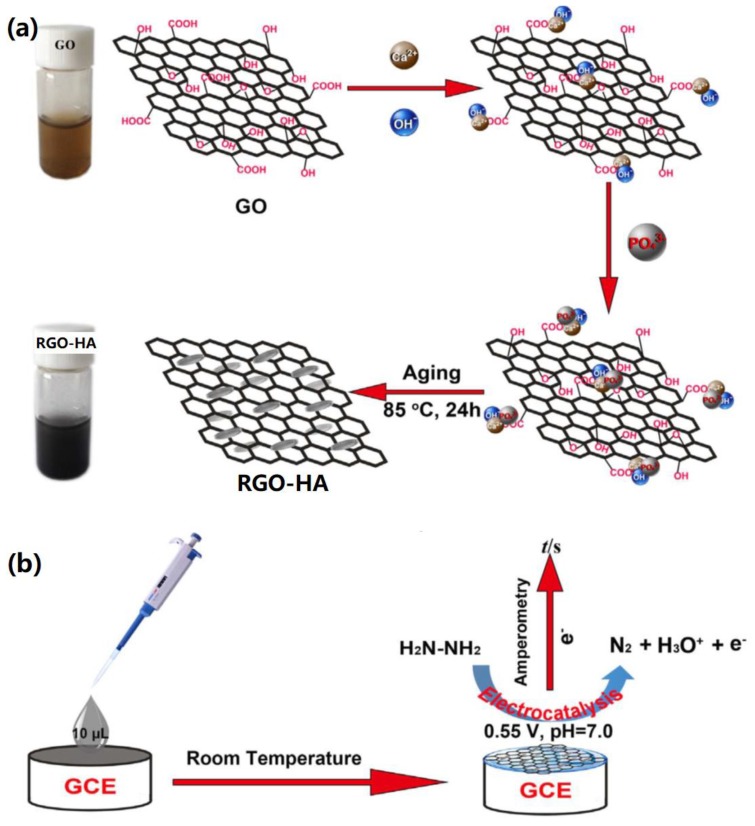
Biomimetic RGO-HA for biosensor application: (**a**) Synthesis mechanism of RGO-HA nanohybrids, (**b**) Sensing mechanism of hydrazine. Reproduced with the permission from ref. [90]. Copyright 2017 Elsevier Ltd.

Recently, Gao et al. designed an electrochemical sensor based on RGO-HA for the oxidation of hydrazine [90]. To fabricate the biosensor architecture, Ca(OH)_2_ was added into the as-prepared GO solution with stirring for 1 h and H_3_PO_4_ solution was then added into the mixed solution, which was aged in an air oven at 85 °C for 24 h to obtain RGO-HA nanocomposites, as shown in Figure 9a. In the next step, the formed RGO-HA nanocomposites were added into 1.0% acetic acid solution containing CS to prepare RGO-HA-CS nanocomposites, which were then dropped onto a GCE to fabricate biosensors (Figure 9b). It was found that RGO-HA materials exhibited fast and significant catalysis towards the oxidation of hydrazine compared to GO and HA. Furthermore, the fabricated biosensors showed a linear detection of 2.5 μM–0.26 mM and 0.26–1.16 mM with a detection limit of 0.43 μM.

In another study, Alam and co-workers developed a selective chemical biosensor based on RGO-HA nanocomposites, and further investigated the electrochemical detection of Bis-phenol A (BPA) by current-voltage (I–V) measurement [91]. The fabricated biosensor exhibited fast and highly sensitive detection towards BPA with a detection limit of 60.0 pM and wide linear detection range from 0.2 nM to 2.0 mM. The excellent electrochemical response properties could be attributed to the higher specific surface area, excellent adsorption ability, high electrocatalytic activity, and biocompatibility of the porous RGO-HA nanomaterials towards BPA.

In addition, based on enzymatic recycling amplification and metal NPs, graphene-HA based electrochemical biosensors have been reported. For instance, Bharath and co-workers fabricated a glucose sensor based on GOx-modified GCE [92]. The as-prepared glucose biosensor showed a linear detection range from 0.1 to 19.2 mM with a detection limit of 0.03 mM. In another case, they also developed a new strategy to synthesize magnetite HA NPs on the edge-carboxylated GO surface and further fabricated a electrochemical biosensor for the detection of 4-nitrophenol (4-NP) through CV and differential pulsed voltammetry measurements [93]. A linear detection range from 0.2 to 994 μM and a low detection limit of 0.27 μM were obtained. In addition, the fabricated biosensor exhibited an excellent electrocatalytic performance, including high sensitivity, good selectivity, and long-term stability.

### 4.5. Antibacterial Effects

Antibacterial effects are crucial for evaluating the potential of implant biomaterials. Graphene-based nanomaterials have been widely utilized for long-term antibacterial applications [94,95,96].

Murugan et al. demonstrated the biomimetic synthesis of HA on GO/CNF nanohybrids for the formation of functional GO/CNF-HA composites, which revealed high mechanical strength and similar characteristics to natural bone [28]. It was found that the synthesized composites exhibited good antibacterial activity against both *Staphylococcus aureus* and *Escherichia coli* (*E. coli*). In a further study, they investigated the biomimetic mineralization of HA on PCL-modified GO substrate [97]. The antibacterial tests towards *Staphylococcus aureus* and *Escherichia coli* indicated that the formed GO/PCL-HA also exhibited a high performance for killing both bacteria due to the release of Mg^2+^ and Zn^2+^ from the biomineralized HA.

**Figure 10:**
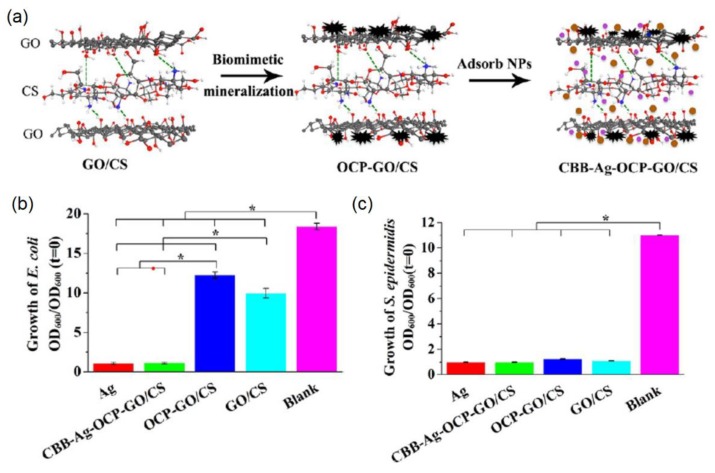
Biomimetic GO-HA nanohybrids for antibacterial application: (**a**) Biomimetic synthesis mechanism; (**b**,**c**) Antibacterial tests towards (**b**) *E. coli* and (**c**) *S. epidermidis*. Reproduced with the permission from [98]. Copyright 2016 American Chemical Society.

Biomimetic GO-HA nanohybrids can also be conjugated with other bacteria-killing components (such as AgNPs and SiO_2_) to improve their antibacterial activity. For instance, Xie and co-workers reported the biomimetic mineralization of CS-functionalized GO (GO/CS) for the formation of HA-like octacalcuim phosphate (OCP) [98]. The mineralized OCP-GO/CS was further utilized to bind with AgNPs and CS-BMP2-BSA (CBB) NPs, which were formed by stabilizing BMP2-encapsulated BSA NPs with CS via the electrostatic interactions, as shown in Figure 10a. The antibacterial tests indicated that the formed CBB-Ag-OCP-GO/CS composites have high antibacterial activity against *E. coli* and *S. epidermidis* with bactericidal ratios of 94% and 91%, respectively. It is clear that the addition of AgNPs to the scaffold enhanced the antibacterial activity of OCP-GO/CS materials. It is interesting that both OCP-GO/CS and GO/CS revealed relative antibacterial activity towards *E. coli* and *S. epidermidis*, as shown in Figure 10b,c. The quantitative analysis demonstrated that the OCP-GO/CS and GO/CS composites contributed about 34% and 47% to the antibacterial effects of materials against *E. coli*, meanwhile 89% and 90% against *S. epidermidis*, respectively. The excellent antibacterial activity of the created CBB-Ag-OCP-GO/CS composites was ascribed to the synergistic effects of AgNPs, GO, and CS.

Besides the material effects for antibacterial activity, drugs with antibacterial activity can also be adsorbed onto/into GO-HA nanohybrids for inhibiting the growth of bacteria. In a typical study, Gholigegloo and co-workers reported the biomimetic synthesis of HA on GO-carnosine conjugates for loading an antibacterial drug, ICG, to enhance the antibacterial effects against *Streptococcus mutans* [67]. It was found that the fabricated GO-carnosine@ICG caused high performance for inhibiting bacterial survival with 86.4%, which proved that carnosine was very effective for killing bacteria. By using a photodynamic therapy, the synthesized GO@ICG, GO-carnosine@ICG, and GO-carnosine/HA@ICG could decreased the counts of bacterial strains to 91.2%, 95.5%, and 93.2%, respectively.

## 5. Conclusions

In summary, we presented recent advances in the biomimetic synthesis of HA crystals on various graphene supports, and further demonstrated and discussed the potential biomedical applications of biomimetic graphene-HA nanohybrids in the fields of drug delivery, cell culture, bone repair and regeneration, biosensors, and antibacterial materials. Previous studies indicated that the functional modification of graphene with various groups, polymers, biomolecules, cells, and others can mediate the biomimetic formation of HA, as well as improve the mechanical strength, bioactivity, and biocompatibility of hybrid materials. A lot of case studies have proven that both graphene supports and biomimetic HA crystals exhibited their unique effects towards improved biomedical applications. We believe that this overview will be helpful for readers to understand the design, synthesis, and mechanisms of biomimetic minerals on graphene supports and explore the biomimetic materials in biomedical engineering, nanotechnology, materials science, analytical science, as well as energy and environmental science. In our opinion, future studies on the following topics could be studied in depth, for instance, the design of 3D porous graphene-HA based materials for cell growth and drug delivery, the combination of NPs like TiO_2_, ZnO, and SiO_2_ with graphene-HA hybrids for improved antibacterial activity, and the synthesis of multi-functional materials for simultaneous performances (such as drug delivery, cancer cells killing, and cell growth promotion), and the development of novel synthesis methods for graphene-HA materials.
